# Current understanding of disease control and its application in patients with chronic rhinosinusitis

**DOI:** 10.3389/fcimb.2023.1104444

**Published:** 2023-06-05

**Authors:** Jiahui Zhou, Fan Yuan, Tianhao Huang, Li Zhu, Dawei Wu

**Affiliations:** ^1^Department of Otolaryngology Head and Neck Surgery, Peking University Third Hospital, Beijing, China; ^2^Department of Otolaryngology Head and Neck Surgery, Beijing Friendship Hospital, Capital Medical University, Beijing, China; ^3^Department of Otolaryngology Head and Neck Surgery, The First Affiliated Hospital of Wenzhou Medical University, Zhejiang, Wen Zhou, China

**Keywords:** sinusitis, treatment outcome, patient outcome assessment, risk factors, prognosis

## Abstract

**Background:**

Disease control is a primary treatment goal for patients with chronic rhinosinusitis (CRS). This study aims to summarize the evaluation parameters of disease control and then identify predictors of poorly controlled CRS.

**Methods:**

A systematic review of the literature was performed on PubMed, Google Scholar, Scopus, and Cochrane databases to identify studies relating to disease control in CRS.

**Results:**

The concept of disease control in patients with CRS involved the longitudinal assessment of the disease state and was also an important goal of treatment. As a metric of the disease state, the disease control reflected the ability to keep disease manifestations within certain limits, the efficacy after treatment, and the impact on quality of life. Validated measurements, such as EPOS2012 criteria, EPOS2020 criteria, Sinus Control Test, and patient/physician-reported global level of CRS control, have been utilized in clinical practice. These existing disease control instruments incorporated various disease manifestations and categorized patients into two (well-controlled and poor-controlled), three (uncontrolled, partly controlled, and controlled), or five (not at all, a little, somewhat, very, and completely) control categories. Eosinophilia, high computerized tomography score, bilateral sinonasal disease, asthma, allergic rhinitis, female gender, aspirin intolerance, revision surgery, low serum amyloid A, and specific T cell subtype would predict poorly controlled CRS.

**Conclusion:**

The concept of disease control and its application were gradually developed in patients with CRS. The existing disease control instruments demonstrated a lack of uniformity regarding the controlled criteria and included parameters.

## Introduction

Chronic rhinosinusitis (CRS) is a chronic inflammatory sinonasal disease that afflicts 10.9% of Europeans (Hastan et al., 2011), 11.9% of Americans (Hirsch et al., 2017), and 8% of Chinese (Shi et al., 2015). Despite maximal medical therapy, up to 60% of patients with CRS have persistent symptoms (Baguley et al., 2014), which are called difficult-to-treat rhinosinusitis or refractory CRS (Fokkens et al., 2012). Furthermore, the revision rate after endoscopic sinus surgery is about 15–20% after five to ten years of follow-up (Alanin and Hopkins, 2020). For patients with chronic rhinosinusitis with nasal polyps (CRSwNP), polyp recurrence after endoscopic sinus surgery is as high as 70% of patients after 18 months of follow-up (DeConde et al., 2017). It seems that patients with CRS need a long-term treatment strategy and there is a growing consensus that the primary treatment goal of CRS is to maintain clinical control (Van der Veen et al., 2017). Identifying the patients with poor disease control states is necessary to guide therapy alterations.

An increasing number of studies are exploring the different aspects of disease control in patients with CRS (Snidvongs et al., 2014). It is important to note that the evaluation parameters of disease control in patients with CRS differ across studies. The 2012 European Position Paper on Rhinosinusitis and Nasal Polyps (EPOS 2012) defined disease control as a disease state free from bothersome symptoms and having a healthy mucosa without the need for systemic medication (Fokkens et al., 2012). It proposed an assessment of CRS disease control based on a combination of symptoms, endoscopy, and systemic medication used (Fokkens et al., 2012). The EPOS2012 criteria have categorized patients into uncontrolled, partly controlled, and controlled CRS. Since the establishment of EPOS2012 criteria for disease control in CRS, several studies have attempted to develop criteria like Physician Global Assessment (PGA), Sinus Control Test (SCT), and patient report of change in global nasal function (Snidvongs et al., 2014; Banglawala et al., 2016; Gray et al., 2017a; Campbell et al., 2018; Sedaghat et al., 2018; McCann et al., 2021; Phillips et al., 2021). In addition, patient/physician-reported global level of CRS control was also developed. These tools classified the patients with CRS into five (Not at all, a little, somewhat, very, and completely) control levels (Gray et al., 2017a; Campbell et al., 2018; Sedaghat et al., 2018; McCann et al., 2021; Phillips et al., 2021). Moreover, several recent studies have explored the predictors of poor disease control, which help to guide for escalation of the CRS treatment regimen (Van der Veen et al., 2017; Tao et al., 2018; Wang et al., 2019; Lu et al., 2021; Penttilä et al., 2021; Jiang et al., 2022). However, these studies utilized varied components of CRS disease control assessments. A systematic review of the current literature on disease control assessment would lay a foundation for developing a gold standard to assess disease control in CRS. Therefore, this systematic review aimed to summarize characteristics of the evaluation measurements of disease control in patients with CRS and then identify predictors of poorly controlled CRS.

## Materials and methods

### Literature search strategy

An evidence-based systematic review was performed utilizing the Preferred Reporting Items for Systematic Reviews and Meta-Analysis (PRISMA) guidelines. A comprehensive search of PubMed, Scopus, Cochrane databases, and Google Scholar was conducted to identify studies relating to disease control in CRS on July 14, 2022. A combination of terms was used in this review: chronic rhinosinusitis, chronic sinusitis, control, and disease control.

### Study selection

**Figure 1:**
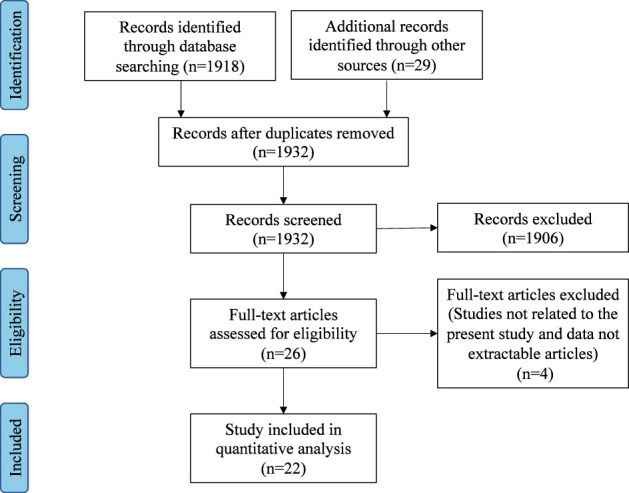
Flow diagram of the study.

Titles and abstracts of all the relevant studies were reviewed by two independent authors (DW and JZ). Included studies addressed the concept, criteria, characteristics, and predictors of disease control in CRS. All included studies were downloaded, and both authors reviewed the full text. **Figure 1** outlines the search strategy and inclusion process used to find relevant studies.

### Data extraction and analysis

Data included the year of publication, study design, concept, clinical application, disease control criteria, intervention, result, and conclusion. After analysis of each article, summary tables were developed. The quality of each article was assessed by the Oxford Center for Evidence-Based Medicine Levels of Evidence categorization (Burns et al., 2011).

## Results

### Include studies

The initial database search identified 1947 articles. After the removal of duplicates and abstract screening, 1906 articles were excluded. A total of 26 articles underwent a full-text assessment for eligibility, and 22 articles met the final inclusion criteria for systematic review.

### Different disease control measures in patients with CRS

A total of thirteen disease control measures in patients with CRS in seventeen studies were summarized, including EPOS2012 criteria, EPOS2020 criteria, Nasal Obstruction Systemic medication used Endoscopic inflammation (NOSE), physician report of condition, PGA, patient’s self-assessment of overall CRS disease control, SCT, patient report of change in global nasal function, patient/physician-reported global level of CRS control with five control levels, Visual analog scale (VAS), Sino-Nasal Outcome Test 22 (SNOT-22) scores, and controlled CRS measured by the revision ESS and exacerbation of CRS during the follow-up (**Table 1**).

**Table 1:** Different disease control assessments in patients with CRS.

Study	Different criteria of disease control	Controlled	Partly controlled	Uncontrolled
(Snidvongs et al., 2014) (Tao et al., 2018) (Van der Veen et al., 2017) (Wang et al., 2019) (Little et al., 2021)	EPOS2012 criteria	1. No bothersome symptoms; 2. Healthy or almost healthy mucosa; 3. No need for systemic medicine.	1. Experience one or two of the following features: symptoms (persistent nasal blockage, mucopurulent rhinorrhea/postnasal drip, facial pain, impaired smell, sleep disturbance/fatigue); 2. Disease mucosa; 3. The need of systemic medication in the last 3 month.	Three or more features of partly controlled.
(Jiang et al., 2022) (Lourijsen et al., 2022)	EPOS 2020 criteria	1. No bothersome symptoms; 2. Healthy or almost healthy mucosa 3. No need for systemic medicine	1. Experience one or two of the following features: Symptoms (persistent nasal blockage, mucopurulent rhinorrhea/postnasal drip, facial pain, impaired smell, sleep disturbance/fatigue); 2. Disease mucosa; 3. The need of rescue treatment in last 6 months.	Three or more features of partly controlled.
**(a).**(Snidvongs et al., 2014)	NOSE: Nasal Obstruction, Systemic medication used, and Endoscopic inflammation	1. No bothersome symptoms; 2. Healthy or almost healthy mucosa; 3. No need for systemic medicine to control disease.	1. Nasal obstruction; 2. Disease mucosa; 3. Short systemic medication in the past 3 months.	Either ‘‘both symptom and endoscopy’’ criteria or long systemic medication.
**(b).**(Snidvongs et al., 2014)	Physician report of condition	Normal mucosa regardless of symptoms	Symptoms with correlating endoscopic features.	Systemic medication and poor progress.
(Banglawala et al., 2016)	Physician Global Assessment (PGA) was based on the history, Lund-Kennedy score, and Lund- Mackay score.	1	2	3
(Phillips et al., 2022)	Patient’s self-assessment of overall CRS disease control	1	2	3
(Banglawala et al., 2016) (Kohli et al., 2017) (Little et al., 2021)	Sinus Control Test (SCT): Sinus symptoms (nasal obstruction, nasal discharge), daily life impact and treatments	0 to 3	4 to 11	12 to 16
(Snidvongs et al., 2014)	Patient report of change in global nasal function (a 13-point Likert-type scale).	+4 to +6	+1 to +3	-6 to 0
(Banglawala et al., 2016) (Gray et al., 2017a) (Campbell et al., 2018) (Sedaghat et al., 2018) (McCann et al., 2021) (Phillips et al., 2021)	Patient report of the global level of CRS control	Completely, very	Somewhat, a little	Not at all
(Sedaghat et al., 2018)	**Physician report of the global level of CRS control:** based on the SNOT-22 score, endoscopy score, the number of sinus infections, CRS-related antibiotics, CRS-related oral corticosteroid courses and missed days of work or school due to CRS in the preceding 3 months as reported by the patient.	Completely, very	Somewhat, a little	Not at all
(Kosak et al., 2015)	Visual analogue scale (VAS)	VAS<5	–	VAS≥5
(Gray et al., 2017b)	SNOT-22	well-controlled<35	–	poor-controlled ≥ 35
(Lilja et al., 2021)	Disease control was measured by using the revision ESS and exacerbation of CRS.	–	–	revision ESS and need for rescue/advanced therapy in the follow-up

EPOS, European Position Paper on Rhinosinusitis and Nasal Polyps; NOSE, Nasal Obstruction, Systemic medication used, and Endoscopic inflammation; PGA, Physician Global Assessment; SCT, Sinus Control Test; CRS, Chronic rhinosinusitis; SNOT-22, Sino-Nasal Outcome Test 22; VAS, Visual analog scale, ESS Endoscopic sinus surgery.

Five studies reported EPOS2012 criteria (Snidvongs et al., 2014; Van der Veen et al., 2017; Tao et al., 2018; Wang et al., 2019; Little et al., 2021), and two studies reported EPOS2020 criteria (Jiang et al., 2022; Lourijsen et al., 2022). Both criteria were based on symptoms, endoscopy, and systemic medication used. Based on validation studies (Snidvongs et al., 2014; Van der Veen et al., 2017; Calus et al., 2019), the EPOS2020 criteria have some improvements over EPOS2012. EPOS2020 criteria recommend using a VAS scale for all symptoms (Fokkens et al., 2020). EPOS2012 focused on systemic medication needed in the last three months, while EPOS2020 focused on rescue treatment in the last six months. In EPOS2012 criteria, uncontrolled CRS needs long-term systematic medication (Fokkens et al., 2012) while patients with uncontrolled CRS still had symptoms after rescue treatment in EPOS2020. Snidvongs et al. (2014) proposed a simpler NOSE system just using nasal obstruction, the systemic medication used, and endoscopic inflammation. Meanwhile, they put forward physician reports of conditions based on the nasal mucosa, symptoms, systemic medication, and progress. The above four measures would divide the patients with CRS into three levels of control, including controlled, partly controlled, and uncontrolled conditions according to the combination of different subjective and objective parameters.

Another two measures including PGA and patient’s self-assessment of overall CRS disease control would directly classify patients with CRS into controlled, partly controlled, and uncontrolled conditions. The treating physicians completed the PGA questionnaire using a 3-point scale. The control rating was based on the history, endoscopic sinus examination, and computed tomography findings. The patients with CRS rated their overall CRS disease control as controlled, partly controlled, and uncontrolled condition. The SCT was utilized in three studies to evaluate disease control in patients with CRS based on the sinus symptoms (nasal obstruction, nasal discharge), daily life impact, and treatments (Banglawala et al., 2016; Kohli et al., 2017; Little et al., 2021). Snidvongs et al. (2014) used a 13-point Likert-type scale to report the change in global nasal function.

Patient self-assessment based on perception or patient report of the global level of CRS control was utilized in six studies where patients were classified into five control levels (Banglawala et al., 2016; Gray et al., 2017a; Campbell et al., 2018; Sedaghat et al., 2018; McCann et al., 2021; Phillips et al., 2021). Similarly, Sedaghat et al. (2018) used physician reports of the global level of CRS control based on the SNOT-22 score, endoscopy score, the number of sinus infections, CRS-related antibiotics, CRS-related oral corticosteroid courses, and missed days of work or school due to CRS in the preceding three months as reported by the patient. Moreover, the patients were divided into five control levels. Kosak, et al (Kosak et al., 2015). used VAS to assess disease control in patients with CRS. A VAS score of less than 5 indicated a well-controlled condition, and a VAS score of more than 5 indicated an uncontrolled condition. Similarly, Gray et al. (2017b) used scores of SNOT-22 to distinguish between patients with well or poor-controlled CRS symptoms. Lilja et al. (2021) identified patients with a revision of ESS and the need for rescue/advanced therapy in the follow-up as uncontrolled CRS.

### Characteristics of different disease control measures

We summarized the characteristics of different disease control measures in thirteen studies (**Table 2**). Snidvongs et al. (2014) proposed NOSE criteria which were simpler than EPOS 2012 criteria, and found that both EPOS 2012 and NOSE significantly correlated with physicians and patient reports. Furthermore, Van der Veen et al. (2017) found that the levels of control measured by EPOS2012 corresponded to mean total SNOT-22 and short form (36) health survey (SF-36) scores (p < 0.05). Lu et al. (2021) found that increased serum amyloid A (SAA) level was associated with better disease control as measured by EPOS2012 criteria in patients with CRSwNP after ESS.

**Table 2:** Characteristics among different disease control measures and their association with disease burden and clinical parameters.

Study	Design	Patients	Intervention	Different disease control measures	Results	Conclusion
(Snidvongs et al., 2014)	Prospective study	CRS(n=106)	–	1.EPOS2012 criteria 2.NOSE 3.Physician and patient report of condition	**1. Patient’s reports of condition:** nasal obstruction associated with patient’s reports of disease control; **2. Physician report of condition:** endoscopic mucosal inflammation, and thick and/or purulent discharge associated with physician’s reports of disease control; **3.The EPOS 2012 and NOSE:** both EPOS 2012 and NOSE had significant agreement with physician’s and patient’s report.	A new disease control staging system using nasal obstruction, mucosa, and discharge is proposed.
(Van der Veen et al., 2017)	Observational study	CRS underwent FESS (n=560)	–	EPOS 2012 criteria	1. The levels of control corresponded to mean total SNOT-22 and SF-36 scores (*p* < 0.05).	The EPOS 2012 levels of disease control are associated with QOL
(Lu et al., 2021)	Retrospective cohort study	non-ECRSwNP (n=26) VS ECRSwNP(n=22) VS Healthy control(n=10)	–	1. EPOS2012 criteria 2. SAA: Serum Amyloid A, Controlled: high SAA (≥114.9 ng/mL), Uncontrolled: low SAA (<114.9 ng/mL).	The SAA level was significantly higher in polyp tissues of the disease-controlled patients than those of the partly controlled and uncontrolled (*p* < 0.001 and 0.01, respectively)	Increased tissue SAA levels is associated with a better prognosis in CRSwNP after ESS.
(Banglawala et al., 2016)	Prospective cohort study	CRS(n=50)	–	1. PGA: Physician Global Assessment together with the history, Lund-Kennedy score, and Lund- Mackay score. 2. SCT: Sinus Control Test. 3. Patient’s self-assessment base on perception.	1.Cohen’s kappa for the agreement between the PGA and SCT of classification was 0.68. 2. The total SNOT-22 scores were significantly correlated with the PGA (*p*<0.001) and patient’s self-assessment of their disease (*p*<0.001).	1. The SCT is a simple, patient generated questionnaire that can measure the control of CRS without requirement of endoscopy or CT evaluation. 2. The SNOT-22 scores were highly associated the severity of disease indicated by physician or patients.
(Kohli et al., 2017)	Prospective non-randomized case series	CRS(n=77)	ESS	SCT	1. Global SCT scores of patients undergoing ESS improved from 8.9 ± 3.8 to 4.6 ± 3.5 (p <0.001). 2. Change in SCT score and change in SNOT-22 score after surgery were significantly correlated (*p <*0.001).	The SCT is responsive to surgical intervention and a reliable tool to monitor changes in CRS control levels.
(Little et al., 2021)	Prospective cohort study	CRS underwent ESS(n=218)	ESS	SCT	1. Mean SCT score improved from 8.9 ± 3.5 to 4.3 ± 3.7 postoperatively (*p*<0.001). 2. Change in SCT score correlated independently with change in SNOT-22(*p*<0.001) and endoscopy scores(*p*<0.001).	1.The SCT provides information complementary to existing patient-reported and objective measures of disease severity. 2. Improvement in disease control following ESS as measured by the SCT correlated with improvements in SNOT-22 and endoscopy scores.
(Gray et al., 2017a)	Prospective cross-sectional study	Adults CRS(n=166)	–	Patients-reported symptom control.	1. Patient’s self-reported level had a statistically significant correlation with SNOT-22 (*p* < 0.001). 2. Higher EQ5D-VAS scores were associated with “Very” (*p* = 0.003) and “Completely” (*p* = 0.014) compared to “Not at all”, associated with “A little” (*p* = 0.024), “Somewhat” (*p* = 0.049), “Very” (*p* = 0.002) and “Completely” (*p* < 0.001).	Higher levels of patient-reported CRS symptom control are associated with better QOL.
(Gray et al., 2017b)	Cross-sectional study	CRS(n=202)	–	1.Patients-reported symptom control. 2.SNOT-22: poor-controlled and well-controlled.	SNOT-22 was negatively associated with patient-reported CRS symptom control (*p* < 0.001).	SNOT-22 score is associated with how well patients feel their CRS symptomatology is controlled.
(Phillips et al., 2021)	Prospective longitudinal study	CRSwNP (n=105) VS CRSsNP (n=195) All(n=300)	–	Patient-reported disease control	1. At enrollment and follow-up timepoints, patient-reported CRS disease control was significantly correlated with SNOT-22 and EQ5D- VAS scores. 2. The change in patient-reported CRS disease control was significantly correlated with change in SNOT-22 and change in EQ5D-VAS scores. 3. There was significant cross-sectional and longitudinal correlation between patient-reported control and all SNOT-22 subdomain scores.	Patient-reported CRS disease control is a valid measure of CRS disease burden and general QOL.
(Campbell et al., 2018)	Prospective cross-sectional cohort study	CRS(n=200)	–	Patients-reported symptom control	CRS symptom control classified as “not at all” was associated with 11 days of lost productivity due to CRS on univariate analysis (*p* < 0.001) and 8 days of lost productivity on multivariate analysis (*p* = 0.011).	Patient-perceived control of CRS symptoms is independently associated with lost productivity.
(Sedaghat et al., 2018)	Cross-sectional study	CRS(n=209)	–	1.Patients-reported global control. 2.Rhinologists-rated global control.	1. Physician-rated CRS control was moderately correlated with patient-reported CRS control (*p* < 0.001). 2. Patient-reported global CRS control was associated with SNOT-22 (*p*< 0.001) and nasal subdomain score (*p*= 0.006) in a multivariable regression model. 3. Physician-rated CRS control was associated with SNOT-22 score (*p*< 0.001), number of acute bacterial CRS exacerbations (*p*=0.014), number of CRS-related oral corticosteroid courses taken in the last 3 months (*p*=0.012), nasal (*p*<0.001), sleep (*p*=0.001) and otologic/facial pain (*p*=0.012)	1.Patients and physicians use different criteria to determine the level of CRS control. 2.Patients consider primarily nasal symptoms while physicians include nasal and extra-nasal symptoms of CRS in determining CRS control. 3.Physicians also independently consider acute bacterial CRS exacerbations, and CRS-related oral corticosteroid.
(Walker et al., 2022)	Qualitative phenomenological study	–	–	Patient perspectives: controlled And uncontrolled.	Three recurring themes determined from patients: 1. use of the terminology “control” adequately represents this phenomenon. 2.components of control could be classified into four main themes relating to symptomatology, exacerbation of comorbid disease, quality of life, acute exacerbations. 3. Patients who deem their CRS is uncontrolled are more willing to escalate their treatment.	1. CRS patients consider their daily symptoms, the severity and frequency of CRS exacerbations, impact on quality of life as well as exacerbation of comorbid disease when thinking about disease control. 2.Disease control is a goal of treatment for patients and uncontrolled disease motivates patients to seek further treatment.
(Millarelli et al., 2022)	Retrospective cohort study	NSAID-ERD (n = 204) VS without NSAID-ERD (n = 520) All CRSwNP (n=724)	Dupilumab versus placebo	EUFOREA definition: Uncontrolled severe CRSwNP	1. At Week 24, least squares mean treatment differences demonstrated significant improvements in nasal polyp score, NC, Lund–Mackay computed tomography, SNOT-22, TSS, rhinosinusitis severity visual analog scale, PNIF, six-item Asthma Control Questionnaire score, smell in patients with NSAID-ERD (*p* < 0.001). 2. Treatment comparisons demonstrated significantly greater improvements with dupilumab in patients with versus without NSAID-ERD for NC (*p* = 0.004), SNOT-22 (*p* = .031), TSS (*p* = 0.043), and PNIF (*p* = 0.0123).	1. Dupilumab was well tolerated in uncontrolled severe CRSwNP patients with/without NSAID-ERD. 2. Dupilumab significantly improved objective measures and patient-reported symptoms to a greater extent in the presence of comorbid NSAID-ERD than without.

CRS, Chronic rhinosinusitis; EPOS, European Position Paper on Rhinosinusitis and Nasal Polyps; NOSE, Nasal Obstruction, Systemic medication used, and Endoscopic inflammation; FESS, Functional endoscopic sinus surgery; SNOT-22, Sino-Nasal Outcome Test 22; SF-36, Short form (36) health survey; QOL, Quality of life; SAA, Serum Amyloid A; CRSwNP, Chronic rhinosinusitis with nasal polyps; ESS, Endoscopic sinus surgery; PGA, Physician Global Assessment; SCT, Sinus Control Test; EQ5D-VAS, 5-dimension EuroQol general health questionnaire from which the visual analogue scale; CRSsNP, Chronic rhinosinusitis without nasal polyps; NSAID-ERD, non-steroidal anti-inflammatory drug exacerbated respiratory disease.

Banglawala, et al (Banglawala et al., 2016). proposed PGA and SCT to measure the control of CRS and found that SCT correlated with PGA. In addition, SNOT-22 scores were significantly associated with disease control indicated by physicians or patients. Furthermore, both Kohli et al. and Little et al (Kohli et al., 2017; Little et al., 2021). found that SCT scores decreased postoperatively, and change in SCT score was significantly correlated with change in SNOT-22 score and endoscopy score. It was demonstrated in two studies that higher levels of patient-reported disease control were associated with better quality of life as measured by SNOT-22 or 5-dimension EuroQol general health questionnaire from the visual analogue scale (EQ-5D VAS) (Gray et al., 2017b; Phillips et al., 2022). Besides, Campbell et al. (2018) found that a low level of patient-reported symptom control was independently related to lost productivity.

Sedaghat, et al (Sedaghat et al., 2018). found that patients and physicians utilized different criteria to determine the level of CRS control. Specifically, nasal and extra-nasal symptoms, acute bacterial CRS exacerbations, and CRS-related oral corticosteroids were associated with physician-rated CRS control, while primarily nasal symptoms were associated with patient-reported global CRS control. Both the CRS control rated by patients and rhinologists had a significant association with SNOT-22 (Sedaghat et al., 2018). Furthermore, Walker et al. (2022) reported that patients would consider daily symptoms, CRS exacerbations, quality of life, and exacerbations of comorbid disease when talking about disease control and uncontrolled disease motivated patients to seek further treatment. Millarelli et al. (2022) defined uncontrolled chronic rhinosinusitis with nasal polyps (CRSwNP) when the patients with CRSwNP presented with persistent or recurring CRSwNP despite long-term intranasal corticosteroid (INCS) and having received at least one course of systemic corticosteroids in the preceding two years and/or previous sinonasal surgery. They also defined severe CRSwNP based on the bilateral CRSwNP with a nasal polyp score (NPS) of ≥4, and persistent symptoms despite long-term INCS with the need for add-on treatment. In this study, the high efficiency of dupilumab in patients with uncontrolled severe CRSwNP patients was demonstrated.

### Predictors of poor disease control in patients with chronic rhinosinusitis

We further summarized the predictors of poor disease control in patients with CRS (**Table 3**). Five studies identified poor disease control in patients with CRS based on EPOS2012, and risk factors included tissue or blood eosinophilia, high CT score, bilateral disease, asthma, and allergic rhinitis. Furthermore, tissue eosinophil ratio >0.206, blood eosinophil ratio >0.025, Lund-Mackay score≥ 15, CT ethmoid score≥maxillary score, female gender, aspirin intolerance, and revision Functional endoscopic sinus surgery (FESS), low serum amyloid A (SAA) were independently associated with the poor disease control (Van der Veen et al., 2017; Tao et al., 2018; Lu et al., 2021). Additionally, Wang et al. (2019) found that concordant blood and tissue eosinophilia can predict poor control better than isolated blood or tissue eosinophilia. Tao et al. (2018) found that prediction models based on tissue or blood eosinophil ratio together with CT score had significantly different uncontrolled levels.

**Table 3:** Predictors of poor disease control in patients with chronic rhinosinusitis.

Study	Design	Patients	Intervention	Different disease control measures	Results	Conclusion
(Tao et al., 2018)	Retrospective case series	CRS underwent ESS(n=136)	–	EPOS 2012 criteria	1. Univariate regression models revealed the risk factors for uncontrolled CRS: tissue eosinophilia, blood eosinophilia, high CT score, bilateral disease, asthma, and allergic rhinitis. 2. Multiple regression models found tissue eosinophil ratio >0.206 (*p* = 0.001) or blood eosinophil ratio >0.025 (*p* = 0.003), Lund-Mackay score≥ 15 (*p* < 0.001) and CT ethmoid score≥maxillary score (*p*=0.037) were independent risk factors.	Simplified and efficient prediction models based on tissue or blood eosinophil ratio and CT score had significantly different uncontrolled levels.
(Van der Veen et al., 2017)	Observational study	CRS underwent FESS (n=560)	–	EPOS 2012 criteria	Female gender (*p* = 0.032), aspirin intolerance (*p* = 0.039) and revision FESS (*p* = 0.002) were associated with higher prevalence of uncontrolled CRS.	Female gender, aspirin intolerance and revision FESS were associated with higher prevalence of uncontrolled CRS.
(Lu et al., 2021)	Retrospective cohort study	non-ECRSwNP (n=26) VS ECRSwNP(n=22) VS Healthy control(n=10)	–	1. EPOS2012 criteria 2. SAA: Serum Amyloid A, Controlled: high SAA (≥114.9 ng/mL), Uncontrolled: low SAA(<114.9 ng/mL).	1. The SAA level was significantly higher in polyp tissues of the disease-controlled patients than those of the partly controlled and uncontrolled (*p* < 0.001 and 0.01, respectively). 2. ROC curve analysis revealed that a cut-off value of 114.9 ng/mL for the tissue SAA level predicted the patients with disease-controlled status with 93.33% sensitivity and 63.64% specificity (AUC = 0.8727, p < 0.001).	1. Measurements of SAA in polyp tissues identify the CRSwNP patients with disease-controlled status after ESS. 2. Increased tissue SAA levels is associated with a better prognosis in CRSwNP after ESS.
(Wang et al., 2019)	Retrospective study	BT-high (n=57) VS B-high (blood eosinophil≥0.3×109/L, n=22) VS T-high (tissue eosinophils≥10%, n=28) VS BT-low (n=76) All CRSwNP (n=183)	–	EPOS2012 criteria	1. Multiple logistic regression models found blood eosinophil count and tissue eosinophil percentage were independently associated with increased risk for poor disease control after adjustments for covariates related to poor treatment outcome. 2. Subjects with concordant blood and tissue eosinophilia had a higher risk for poor disease control than those with isolated blood or tissue eosinophilia.	Concordant blood and tissue eosinophilia relates to a higher likelihood of poor disease control than isolated blood or tissue eosinophilia
(Tao et al., 2018)	Retrospective case series	CRS underwent ESS(n=136)	–	EPOS2012 criteria	The pathological model was based on tissue eosinophil ratio and CT score (AUC=0.849), and the clinical model was based on blood eosinophil ratio and CT scores (AUC=0.828), both different classifications had significantly different uncontrolled levels (P <.001).	Simplified and efficient prediction models based on tissue or blood eosinophil ratio and CT score had significantly different uncontrolled levels.
(Jiang et al., 2022)	Retrospective and nonconcurrent cohort study	CRSwNP underwent FESS(n=325)	Topical corticosteroids-budesonide nasal spray (256ug/day for 6 months), and intranasal budesonide suspension (1mg/day for 4 weeks) after surgery.	EPOS2020 criteria	1. In the training cohort, the AUC: AS 0.665 (0.593-0.737), AR 0.595 (0.52.8-0.66.2), PBEC 0.658(0.586-0.730), TER 0.684(0.616-0.752). 2.The nomogram showed the highest accuracy with an AUC of 0.760 (95% CI, 0.688-0.830).	1. The asthma, AR, TER, PBEC had significantly affected the disease control of CRS after surgery.
(Penttilä et al., 2021)	Retrospective study	CRSwNP underwent surgery (n = 137)	–	EPOS2020 criteria: controlled And uncontrolled.	The best predictive model was obtained by a sum of baseline (1) blood eosinophilia ≥ 250 cells/μl and/or NP eosinophilia ≥ 30% (Eos), (2) asthma/NERD, and (3) ≥ 1 OCS/year (1 point for each risk factor, AUC > 0.75, *p* < 0.01).	The sum model of eosinophilia, asthma/NERD and OCS had a good predictive potential for uncontrolled CRSwNP.
(McCann et al., 2021)	Prospective, cross-sectional	CRSwNP (n=102) VS CRSsNP(n=206) All(n=308)	–	Patient-reported CRS symptom control	1. On univariate association, CRS symptom control was significantly associated with nasal obstruction, hyposmia, and drainage in both CRSwNP and CRSsNP (P <.05 in all cases). 2. Using multivariable regression to account for all nasal symptoms, only nasal obstruction and nasal discharge scores (but not hyposmia) were significantly associated with CRS symptom control.	1. Hyposmia rarely occurs without nasal obstruction or nasal drainage. 2. Hyposmia was not associated with patient-reported CRS symptom control when accounting for the burden of nasal obstruction and drainage.
(Phillips et al., 2021)	Prospective longitudinal study	CRSwNP (n=105) VS CRSsNP (n=195) All(n=300)	–	Patient-reported disease control	1. A SNOT-22 score of ≤25 points, or an EQ-5D VAS score of ≥77 was predictive of having well – (i.e. ‘very’ or ‘completely’) controlled CRS.	Patient-reported CRS disease control is a valid measure of CRS disease burden and general QOL.
(Gray et al., 2017b)	Cross-sectional study	CRS(n=202)	–	1. Patients-reported symptom control. 2. SNOT-22: poor-controlled and well-controlled	SNOT-22 score of 35 maximized the sensitivity and specificity of detecting patients who felt that their CRS symptoms were poorly controlled.	SNOT-22 score can be used to accurately distinguish patients with poor vs well-controlled CRS symptoms.
(Phillips et al., 2022)	Cross-sectional study	CRS patients(n=309)	–	1.Patient-rated overall CRS control 2. VAS 3. Binary EPOS descriptive symptom scales	1. Symptom burdens measured by VAS, binary descriptive EPOS scale and SNOT-22 were associated with worsening CRS disease control reported by participants. 2. When considering all symptom data simultaneously, a VAS score>3.5 strongly predicted the uncontrolled option on the corresponding binary descriptive EPOS symptom scale for all 5 symptoms. 3. The predictive ability of VAS for rhinorrhea/postnasal drip was disparately worse than the other 4 symptoms.	1. A VAS symptom score of >3.5 translates to the uncontrolled. 2. The rhinorrhea/postnasal drip descriptive symptom scale translates disparately worse to VAS scores and may be considered for revision in future criteria.
(Kosak et al., 2015)	Randomised controlled trial	CRSwNP(n=18)VS CRSsNP (n=13) All untreated CRS(n=31)	Medical or surgical treatment	1. VAS: Well-controlled: VAS<5, Uncontrolled: VAS≥5 2.T cell: CRSwNP group: Well-controlled: Th17 CD4 +CCR6+, Uncontrolled: T17 CD4-CD8-CCR6+ cell CRSsNP group: Uncontrolled: Tc CD8+, Well-controlled: Tc CD4-CD8-.	1. CRSwNP group Th17 CD4+CCR6+: Well-controlled vs Uncontrolled (13.28 ± 5.96 vs 7.16 ± 4.89, *p* = 0.046) 2. CRSwNP group double negative Th17 CD4-CD8-CCR6+ cells: Uncontrolled vs Well-controlled (12.20 vs 5.40; *p* = 0.01) 3. CRSsNP group cytotoxic CD3+CD8+ cells: Uncontrolled vs Well-controlled (79.86 ± 7.86 vs 52.95 ± 28.17, *p* = 0.045) 4. CRSsNP group double negative CD4-CD8- cells: Well-controlled vs. Uncontrolled (46.93 ± 28.29 vs 20.70 ± 7.59, *p* = 0.019)	1. The Th17 CD4 +CCR6+ cell to predict well-controlled CRSwNP. 2.Double negative T17 CD4-CD8-CCR6+ cell (also capable of IL-17 production) predicted uncontrolled CRSwNP. 3. In CRSsNP being cytotoxic type of inflammation the Tc CD8+ cell predicted the uncontrolled disease. 4. The double negative CD4-CD8- predicted the well-controlled CRSsNP.

CRS, Chronic rhinosinusitis; EPOS, European Position Paper on Rhinosinusitis and Nasal Polyps; FESS, Functional endoscopic sinus surgery; SAA, Serum Amyloid A; CRSwNP, Chronic rhinosinusitis with nasal polyps; BT-high, Blood and tissue eosinophil-high; B-high, Blood eosinophil-high; T-high, Tissue eosinophil-high; BT-low, Blood and tissue eosinophil-low; AS, Asthma; AR, Allergy rhinitis; PBEC, Peripheral blood eosinophil count; TER, Tissue eosinophil ratio; NERD, non-steroidal anti-inflammatory drug exacerbated respiratory disease; OCS, oral corticosteroid; SNOT-22, Sino-Nasal Outcome Test 22; EQ5D-VAS, 5-dimension EuroQol general health questionnaire from the visual analogue scale; QOL, Quality of life; VAS, Visual analog scale.

EPOS2020 criteria were used in two studies (Penttilä et al., 2021; Jiang et al., 2022). Jiang et al. (2022) found that the presence of asthma, allergy rhinitis (AR), tissue eosinophil ratio (TER), and peripheral blood eosinophil count (PEBC) affected disease control postoperatively, and the sum nomogram showed the highest accuracy (AUC=0.760). Similarly, Penttilä et al. (2021) reported that the sum model of baseline eosinophilia, asthma/non-steroidal anti-inflammatory drug exacerbated respiratory disease (NERD), and oral corticosteroids (OCS) could well predict uncontrolled CRSwNP.

Patient report of five levels of CRS control was used in three studies (Gray et al., 2017a; McCann et al., 2021; Phillips et al., 2021). It is reported that hyposmia was not associated with poor control because hyposmia seldom occurs without nasal obstruction/drainage (McCann et al., 2021). Furthermore, only nasal obstruction and nasal discharge scores were significantly associated with CRS symptom control. Phillips et al. (2021) reported that SNOT-22 scores>25 or EQ-5D VAS scores <77 had the predictive significance of poorly controlled CRS, while another study by Gray, et al (Gray et al., 2017b). showed that SNOT-22 scores of 35 accurately distinguished poor or well-controlled CRS. A recent study by Phillips et al. (2022) utilized three levels of patient-rated overall CRS control and found that VAS symptom scores >3.5 related to the uncontrolled status on the corresponding binary descriptive EPOS symptom scale for all five symptoms, but the rhinorrhea/postnasal drip descriptive symptom scale translates disparately worse to VAS scores. A study by Kosak et al. (2015) defined VAS≥5 as uncontrolled CRS and showed that T17 CD4-CD8-CCR6+ cells predicted uncontrolled CRSwNP while Tc CD8+ cells predicted uncontrolled chronic rhinosinusitis without nasal polyps (CRSsNP).

## Discussion

There is a growing awareness among physicians that poorly controlled CRS remains a chronic disease, and regular assessment of the disease control level of CRS is crucial to maintain long-term effective treatment of CRS (Guo et al., 2021). Currently, the concept of disease control in patients with CRS has been widely accepted (Fokkens et al., 2020; Orlandi et al., 2021). The definition, evaluation methods of disease control, and their application in CRS management are still active areas of study. Thus, a comprehensive understanding of disease control in patients with CRS would facilitate individualized treatment and also help to unify the application and interpretation of the existing evaluation methods.

We summarized the clinical application of disease control in patients with CRS. Disease control in CRS was first defined as a disease state where disease manifestations were limited to a certain extent (Snidvongs et al., 2014; Kohli et al., 2017; Van der Veen et al., 2017; Tao et al., 2018), reflecting the disease burden or disease severity, efficacy after ESS or medical therapy, the prediction of treatment response or prognosis, and the impact on quality of life. It can be inferred that patients with CRS would be classified into different levels of CRS control. Furthermore, the definition of disease control in CRS among the studies involved the longitudinal assessment of the disease state based on symptoms, sinonasal mucosa, and systematic or local medication, which helps to identify uncontrolled or well-controlled CRS at different points in time (Gray et al., 2017b; Sedaghat et al., 2018). The terminology “disease control” (also known as controlled or well-controlled CRS) also represented an important goal of treatment for patients with CRS (McCann et al., 2021; Phillips et al., 2021; Walker et al., 2022), especially in patients with difficult-to-treat CRS. There is no consensus on the criteria of CRS disease control, and critical components of CRS disease control should be explored in future studies.

We next summarized the characteristics of different disease control measures in patients with CRS. EPOS2012 utilized binary symptoms, endoscopy, and systemic medication used to assess disease control, in which patients with CRS were divided into three control levels including controlled, partly controlled, and uncontrolled (Fokkens et al., 2020). Previous studies validated the reliability of EPOS2012 criteria and showed that EPOS2012 related to physicians and patient reports of overall CRS disease control, total SNOT-22, and SF-36 scores (Snidvongs et al., 2014; Van der Veen et al., 2017). However, a study by Van der Veen et al. showed that only 4 of 21 patients who thought themselves had controlled CRS met the criteria of being controlled (Van der Veen et al., 2017), which indicated that EPOS2012 criteria might overrate the proportion of uncontrolled CRS. On this basis, EPOS2020 criteria recommend using a VAS scale for all symptoms, and a VAS score of more than 5 defines their clinical significance (Fokkens et al., 2020). A recent study by Sedaghat et al. also demonstrated that EPOS2020guidelines regularly assess worse CRS control than those assessed by patients (Sedaghat et al., 2022). The binary symptoms criteria and inclusion of nasal endoscopy may contribute to the discordance of EPOS2020 with patient-reported CRS control (Sedaghat et al., 2022).

Apart from the CRS control criteria proposed by EPOS2012 and EPOS2020, alternative tools for the assessment of CRS control have been proposed. A simpler NOSE system just using nasal obstruction, the systemic medication used, and the presence of endoscopic inflammation was proved to be significantly associated with physicians and patient reports of CRS disease control (Snidvongs et al., 2014). Besides, the PGA was based on the history, endoscopic sinus examination, and computed tomography findings and the physicians rated CRS disease control as controlled, partly controlled, and uncontrolled (Banglawala et al., 2016). Based on the sinus symptoms (nasal obstruction, nasal discharge), daily life impact, and treatments, the SCT was proposed, which was proved to have a significant agreement with PGA (Banglawala et al., 2016). Furthermore, SCT scores in postoperative patients significantly decreased, and the change in SCT score was significantly associated with the change in SNOT-22 score and endoscopy score (Kohli et al., 2017; Little et al., 2021). It can be speculated that the SCT provided information about the degrees of CRS control level and was responsive to change after endoscopic sinus surgery, indicating a complementary role of SCT to the existing assessment of disease control in CRS.

Patient/physician-report CRS disease control was widely applied in studies (Snidvongs et al., 2014; Banglawala et al., 2016; Gray et al., 2017b; Campbell et al., 2018; Sedaghat et al., 2018; McCann et al., 2021; Phillips et al., 2022). However, physician-rated CRS control was moderately correlated with patient-reported CRS control (Sedaghat et al., 2018). The difference between patient-rated and physician-assessed disease control levels may suggest that they considered different factors when assessing disease control. Physicians always take symptoms, acute exacerbations, and oral corticosteroids into account, while patients pay more attention to primarily nasal symptoms (Sedaghat et al., 2018). From a clinical point of view, care should be taken to balance the focus of the patient and the physician. The poorly controlled CRS perceived by the patients motivates them to seek further treatment, and physician-assessed disease control levels would directly inform management decisions. Above all, both objective and subjective parameters should be taken into consideration when conducting the evaluation of disease control in patients with CRS.

We also reviewed the predictors of poor disease control in patients with CRS. Tissue or blood eosinophilia, high CT score, bilateral disease, asthma, allergic rhinitis, female gender, aspirin intolerance, and revision FESS were all independent predictors of poor controlled CRS (Van der Veen et al., 2017; Tao et al., 2018; Wang et al., 2019; Penttilä et al., 2021; Jiang et al., 2022), and concordant blood and tissue eosinophilia predicted better than isolated blood or tissue eosinophilia (Wang et al., 2019). Meanwhile, several sum models based on the above factors were demonstrated to have higher prediction accuracy (Tao et al., 2018; Penttilä et al., 2021; Jiang et al., 2022). Furthermore, low SAA was independently associated with poor disease control. Unexpectedly, as a common symptom caused by inflammatory injury (Smith et al., 2021) or obstruction (Rombaux et al., 2016), hyposmia often occurs together with nasal obstruction/rhinorrhea (McCann et al., 2021). Moreover, without nasal obstruction/drainage, hyposmia had no significant association with patients’ perceptions of CRS control level (Snidvongs et al., 2014; Banglawala et al., 2016; McCann et al., 2021).. However, an impaired sense of smell was associated with the patient’s agreement with EPOS2020 guidelines on having “uncontrolled” CRS (Sedaghat et al., 2022). Regarding the T cell subtype, T17 CD4-CD8-CCR6+ cells were associated with poorly controlled CRSwNP, while Tc CD8+ cells predicted poorly controlled CRSsNP (Kosak et al., 2015). Besides, high SNOT-22 scores, low EQ-5D scores, and high VAS symptom scores were also significantly associated with poorly controlled CRS, which reflected high disease burden and poor QOL (Gray et al., 2017a; Phillips et al., 2021; Phillips et al., 2022). These findings on prediction factors would facilitate preventive interventions and upgrade treatment to achieve disease control.

Some meaningful factors have not been studied in CRS disease control but may inform predictions of poorly controlled CRS. Vitamin D is recognized for its anti-inflammation and antiproliferation effects in previous studies (Stokes and Rimmer, 2016). Compared to healthy subjects, patients with CRS presented a lower level of serum vitamin D (Li et al., 2021). Meanwhile, patients with CRSwNP had a significantly lower level of Vitamin D than patients with CRSsNP (Stokes and Rimmer, 2016; Bavi et al., 2019; Li et al., 2021). It can be inferred that serum vitamin D levels were highly associated with CRS phenotypes. More studies are needed to explore the association of low levels of Vitamin D with poor disease control. It is proved that various fungal related components are able induce type 2 inflammation among patients with sinonasal diseases (Tyler et al., 2021; Challapalli et al., 2022). Eosinophilic CRS is characterized by a type 2 immune response (Tyler et al., 2021) and is frequently associated with a poor disease control status. Further studies should clarify the role of the fungus in the uncontrolled patients with CRS.

## Conclusions

As a refractory disease, the concept of disease control and its application were gradually developed, and a longitudinal assessment of disease control in patients with CRS is crucial to achieving a satisfactory outcome. There is currently no consensus on the notion and critical components of CRS disease control. The existing disease control instruments demonstrated a lack of uniformity regarding the controlled criteria and included parameters. Our review of the literature suggests that eosinophilia, high CT score, bilateral disease, asthma, allergic rhinitis, female gender, aspirin intolerance, revision FESS, low SAA, and T cell subtype would predict poorly controlled CRS. For higher reliability and accuracy, more studies are needed to build a consensus on the certain definition and criteria of disease control in patients with CRS, which is based on the combination of subjective parameters and objective indicators.

## Author contributions

JZ and DW drafted the manuscript. FY and TH analyzed the data. LZ reviewed and revised this manuscript. All authors contributed to the article and approved the submitted version.
